# Differential Response of the Photosynthetic Machinery to Fluctuating Light in Mature and Young Leaves of *Dendrobium officinale*

**DOI:** 10.3389/fpls.2021.829783

**Published:** 2022-02-03

**Authors:** Ying-Jie Yang, Qi Shi, Hu Sun, Ren-Qiang Mei, Wei Huang

**Affiliations:** ^1^Kunming Institute of Botany, Chinese Academy of Sciences, Kunming, China; ^2^University of Chinese Academy of Sciences, Beijing, China; ^3^Bio-Innovation Center of DR PLANT, Kunming Institute of Botany, Chinese Academy of Sciences, Kunming, China

**Keywords:** photosynthesis, photosystem I, photoprotection, cyclic electron flow, water-water cycle

## Abstract

A key component of photosynthetic electron transport chain, photosystem I (PSI), is susceptible to the fluctuating light (FL) in angiosperms. Cyclic electron flow (CEF) around PSI and water-water cycle (WWC) are both used by the epiphytic orchid *Dendrobium officinale* to protect PSI under FL. This study examined whether the ontogenetic stage of leaf has an impact on the photoprotective mechanisms dealing with FL. Thus, chlorophyll fluorescence and P700 signals under FL were measured in *D. officinale* young and mature leaves. Upon transition from dark to actinic light, a rapid re-oxidation of P700 was observed in mature leaves but disappeared in young leaves, indicating that WWC existed in mature leaves but was lacking in young leaves. After shifting from low to high light, PSI over-reduction was clearly missing in mature leaves. By comparison, young leaves showed a transient PSI over-reduction within the first 30 s, which was accompanied with highly activation of CEF. Therefore, the effect of FL on PSI redox state depends on the leaf ontogenetic stage. In mature leaves, WWC is employed to avoid PSI over-reduction. In young leaves, CEF around PSI is enhanced to compensate for the lack of WWC and thus to prevent an uncontrolled PSI over-reduction induced by FL.

## Introduction

A typical light condition for plants in nature is the fluctuations of light intensity owing to cloud, wind, and shading from upper leaves and plants (Pearcy, 1990). When light intensity transiently shifts from low to high, photosystem II (PSII) electron flow rapidly increases but CO_2_ assimilation rate increased slowly (Gerotto et al., 2016; Acevedo-Siaca et al., 2020; De Souza et al., 2020; Grieco et al., 2020; Kimura et al., 2020; Yamori et al., 2020), leading to the imbalance between light and dark reactions (Yamori et al., 2016; Slattery et al., 2018). Within the first seconds after light intensity suddenly increase, electrons transported from PSII to photosystem I (PSI) cannot be immediately transported to NADP^+^ because the consumption of nicotinamide adenine dinucleotide phosphate (NADPH) is restricted, resulting in the accumulation of reducing power in PSI as demonstrated by PSI over-reduction (Yamamoto et al., 2016; Wada et al., 2018). Therefore, fluctuating light (FL) can give rise to a risk of PSI photoinhibition in photosynthetic organisms (Suorsa et al., 2012; Kono et al., 2014; Yamamoto and Shikanai, 2019; Storti et al., 2020). As PSI is the key component of photosynthetic electron flow, PSI photoinhibition suppresses CO_2_ fixation and photoprotection (Sejima et al., 2014; Brestic et al., 2015, 2016; Zivcak et al., 2015, 2019; Chovancek et al., 2019, 2021; Shimakawa and Miyake, 2019). In addition, the rate of PSI repair is much shower than that of PSII (Zhang and Scheller, 2004; Zivcak et al., 2015; Lima-Melo et al., 2019). Therefore, plants should protect PSI from damage when exposed to natural FL conditions (Tikkanen et al., 2012; Allahverdiyeva et al., 2015; Ferroni et al., 2020).

The photoprotective mechanisms coping with the FL in photosynthetic organisms is related to the evolutionary process (Ilík et al., 2017). In non-angiosperms, O_2_ photo-reduction catalyzed by flavodiiron proteins is the main regulatory mechanism coping with FL, which is supplemented by cyclic electron flow (CEF) (Gerotto et al., 2016; Jokel et al., 2018; Storti et al., 2019, 2020). Interestingly, the genes of flavodiiron proteins are completely lost in angiosperms (Yamamoto et al., 2016; Ilík et al., 2017). However, CEF pathways, such as proton gradient regulation 5 (*pgr5*) and chloroplast NADH dehydrogenase-like (NDH) pathways, are retained in the most angiosperms to sustain photosynthesis (Takahashi et al., 2009; Johnson, 2011; Yamori et al., 2011; Nishikawa et al., 2012; Yamori and Shikanai, 2016; Shikanai and Yamamoto, 2017; Rantala et al., 2020). *Arabidopsis thaliana* and rice (*Oryza sativa*) mutants lacking *pgr5* and NDH display stronger PSI over-reduction under high light and thus are susceptible to PSI photoinhibition in the FL (Suorsa et al., 2012; Kono et al., 2014; Yamori et al., 2016; Tikkanen et al., 2017; Yamamoto and Shikanai, 2019). In particular, *pgr5* seedlings died when grown under FL owing to an uncontrolled PSI photoinhibition (Suorsa et al., 2012). After light intensity abruptly increases, CEF is highly stimulated in model C3 plants *Arabidopsis* and tobacco (*Tabacum nicotiana*) (Kono et al., 2014; Yang et al., 2019a). Such activation of CEF favors the proton gradient (ΔpH) formation, which is essential for the PSI photoprotection by slowing down plastoquinone oxidation at the cytochrome b6f (Cyt b6f) and enhancing the electron downstream of PSI (Armbruster et al., 2017). However, the activation of CEF cannot immediately consume the excess electrons in PSI and has some delay in alleviating PSI over-reduction. In addition, a pseudo-CEF in angiosperms, called water-water cycle (WWC), can rapidly consume the excess electrons in PSI and thus protects PSI from damage under FL more efficiently than CEF in angiosperms (Alric and Johnson, 2017; Huang et al., 2019b; Yang et al., 2019b,^2020^; Sun et al., 2020). During WWC, electrons transported from H_2_O to PSI are consumed by photo-reduction of O_2_. The resulting reactive oxygen species (ROS) are scavenged by superoxide dismutase and ascorbate peroxidase (Asada, 1999). This process not only consumes excess reducing power in PSI but also enhance ΔpH formation (Asada, 2000; Rizhsky et al., 2003; Hirotsu et al., 2004; Roberty et al., 2014). Moreover, PSI redox state is always affected by electron flow from PSII. Once PSII activity is downregulated, FL-induced PSI over-reduction can be alleviated (Tikkanen et al., 2014; Suorsa et al., 2016; Terashima et al., 2021). Therefore, the strategies employed to cope with FL vary among angiosperms.

In addition to species difference, the response of PSI to FL can be affected by leaf ontogenetic stage. In field-grown *Cerasus cerasoides* plants, mature leaves displayed more severe PSI over-reduction than young leaves after light increased, leading to stronger FL-induced PSI photoinhibition in mature leaves (Yang et al., 2019c). By comparison, in the crassulacean acid metabolism (CAM) plant *Bryophyllum pinnatum*, FL induced more severe PSI over-reduction and PSI photoinhibition in young leaves than mature leaves (Yang et al., 2019b). These contrasting reports indicated that young and mature leaves might display different responses of PSI to FL. Furthermore, the regulatory mechanisms related to PSI photoprotection significantly differed between young and mature leaves in *C. cerasoides* and *B. pinnatum.* In C3 plant *C. cerasoides* young leaves, the downregulation of PSII activity and enhancement of CEF finely protected PSI under FL (Yang et al., 2019c). In CAM plant *B. pinnatum*, WWC was operational in mature leaves but was negligible in young leaves (Yang et al., 2019b). In the facultative CAM plant *Dendrobium officinale*, WWC was functional in PSI photoprotection under FL in mature leaves (Yang et al., 2020, 2021b; Huang et al., 2021; Sun et al., 2021). CAM plants usually experience drought stress under natural habitats. When CO_2_ assimilation is restricted under drought stress (Zhou et al., 2007; Zhu et al., 2009; Zivcak et al., 2014; Dąbrowski et al., 2019), WWC is a potential protective valve for excess energy (Zivcak et al., 2013; Yi et al., 2014). Therefore, WWC might be a common strategy employed by obligatory and facultative CAM plants to cope with the drought stress and FL. However, it is unclear whether the response of PSI to FL and the related strategies for photosynthetic regulation are also affected by the leaf ontogenetic stage in *D. officinale*. Specifically, we hypothesize that the relative importance of CEF and WWC is dependent on leaf age in *D. officinale*.

*Dendrobium officinale* is a perennial herb that belongs to the *Dendrobium* of Orchidaceae. It is a traditional and extremely precious Chinese herb with high medicinal value. Recently, *D. officinale* has been widely cultivated to meet the market requirement. However, little is known about the characteristics of photosynthetic physiology. In this study, we measured the chlorophyll fluorescence and P700 signals in young and mature leaves of *D. officinale*. This study aimed to: (1) examine whether the response of PSI to FL differs between young and mature leaves, and (2) assess whether the mechanisms of photosynthetic regulation under FL is influenced by the leaf ontogenetic stage. Our results indicated that, when exposed to FL, PSI over-reduction was observed in young leaves but disappeared in mature leaves. The WWC activity contributed to the rapid consumption of excess reducing power in mature leaves. In contrast, CEF was enhanced in young leaves to compensate for the lack of WWC activity and to adjust PSI redox state under FL.

## Materials and Methods

### Plant Materials and Growth Conditions

Tissue-cultured seedlings of *D. officinale* Kimura et Migo plants came from the Kunming Institute of Botany, Chinese Academy of Sciences and were cultivated in this place. All plants were grown in a greenhouse with moderate relative air humidity (60–70%) and 40% of full sunlight. Light condition is controlled using non-woven shade net, and the maximum light intensity at daytime is approximately 800 μmol photons m^–2^ s^–1^. To avoid water or nutrition stresses, plants were watered every day and fertilized by compound fertilizer. Young (flushed within 20 days) and mature (flushed 2 months ago) leaves were used for photosynthetic measurements that were conducted in late July 2021.

### Chlorophyll Content Measurement *in vivo*

The relative content of chlorophyll per unit leaf area was measured using a two-wavelength-type, handy chlorophyll meter (SPAD-502 Plus; Minolta, Tokyo, Japan).

### Redox Changes of P700 After Transition From Dark to Actinic Light

The redox change of P700 after transition from dark to actinic light was measured using a Dual-PAM 100 measuring system (Heinz Walz, Effeltrich, Germany). After dark adaptation for at least 60 min to inactivate the Calvin–Benson cycle, intact leaves were illuminated at 1,809 μmol photons m^–2^ s^–1^ under atmospheric air condition at approximately 25°C (Ilík et al., 2017).

### Photosystem I and II Measurements

In the morning (9–11 a.m.), PSI and PSII parameters were measured on intact uncut leaves at approximately 25°C using a Dual-PAM 100 measuring system (Heinz Walz, Effeltrich, Germany) (Schreiber and Klughammer, 2008). The initial PSI and PSII parameters were measured after dark-adaptation for 30 min. A 635-nm light-emitting diode array was used as actinic light for illumination. After photosynthetic induction at 923 μmol photons m^–2^ s^–1^ for 15 min, leaves were illuminated at a low light of 59 μmol photons m^–2^ s^–1^ for 5 min. Afterward, leaves were exposed to FL alternating between 1,809 and 59 μmol photons m^–2^ s^–1^. During two cycles of low/high light, PSI and PSII parameters were measured. PSI parameters were calculated as follows: the quantum yield of PSI photochemistry, $\text{Y}\,\left(\text{I}\right)=\left(P_{\text{m}}^{{}^{\prime }}-P\right)\,/\,P_{\text{m}}$; the oxidation ratio of P700, Y (ND) = *P*/*P*_m_; and the extend of PSI over-reduction, $\text{Y}\,\left(\text{NA}\right)=\left(P_{\text{m}}-P_{\text{m}}^{{}^{\prime }}\right)\,/\,P_{\text{m}}$. The PSII parameters were calculated as follows: the quantum yield of PSII photochemistry, $\text{Y}\,\left(\text{II}\right)=\left(F_{m}^{{}^{\prime }}-F_{s}\right)\,/\,F_{m}^{{}^{\prime }}$; the quantum yield of non-regulatory energy dissipation in PSII, Y (NO) = *F*_*s*_/*F*_*m*_; and the quantum yield of non-photochemical quenching in PSII, Y(NPQ) =  1−Y(II)−Y(NO).

The photosynthetic electron transport rates (ETRs) through PSI and PSII were calculated as follows: electron transport rate through PSI (ETRI) = PAR × Y(I) × 0.84 × 0.5; electron transport rate through PSII (ETRII) = PPFD × Y(II) × 0.84 × 0.5. PPFD is the photosynthetically active radiation; 0.84, the light absorption of incident irradiance; 0.5, the fraction of absorbed light reaching PSI or PSII. The apparent rate of CEF was estimated by subtracting ETRII from ETRI (Zivcak et al., 2013; Hepworth et al., 2021). These ETR calculations based on assumptions that the light absorption and the fraction of absorbed light reaching PSI or PSII did not differ between young and mature leaves.

### Statistical Analysis

All data are displayed as means of five leaves from five independent plants. A *T*-test was used to determine whether significant differences existed between different treatments (α = 0.05).

## Results

### The Activity of Water-Water Cycle Differed Between Young and Mature Leaves

**FIGURE 1:**
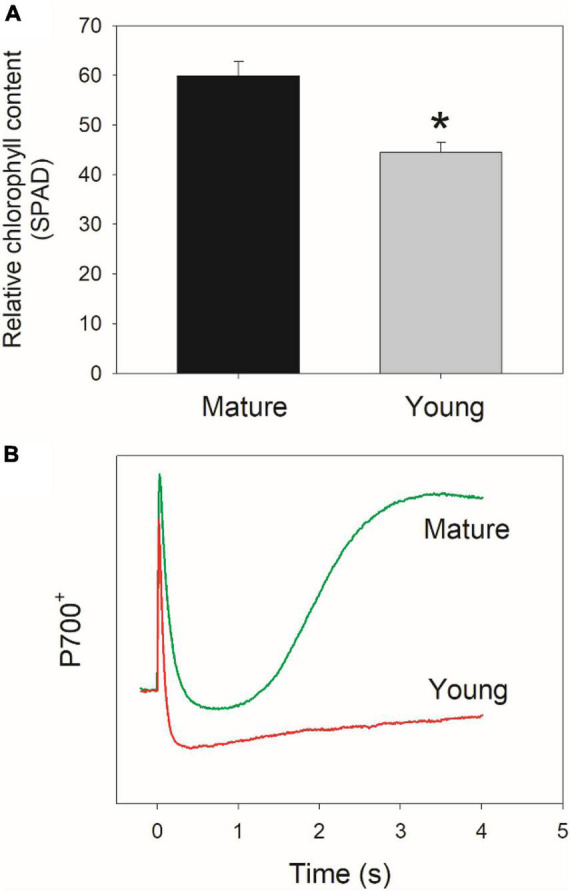
**(A)** Relative chlorophyll content (measured by SPAD) in mature and young leaves of *Dendrobium officinale*. **(B)** Redox kinetics of P700 after shifting from dark to actinic light (1,809 μmol photons m^–2^ s^–1^) in mature and young leaves. Data are means ± SE (*n* = 5). Asterisk indicates a significant difference between mature and young leaves.

For plants of *D. officinale*, the young leaves are reddish and the mature leaves are green. The relative chlorophyll content, as demonstrated by SPAD value, was significantly lower in young leaves than mature leaves (Figure 1A). After shifting from dark to 1,809 μmol photons m^–2^ s^–1^, mature leaves showed the rapid re-oxidation of P700 in 3 s (Figure 1B). However, such rapid P700 re-oxidation was not observed in young leaves (Figure 1B). Many previous studies have indicated that this rapid re-oxidation of P700 in angiosperms is caused by the fast outflow of electrons from PSI to O_2_ mediated by the WWC activity (Shirao et al., 2013; Huang et al., 2019b,^2021^; Sun et al., 2020; Yang et al., 2020). Therefore, WWC activity was present in mature leaves but was lacking in young leaves.

### Photosynthetic Performances Upon Transition From Low to High Light Differed Between Young and Mature Leaves

Under FL, the responses of PSI and PSII to a sudden increase in illumination significantly affected the extent of photoinhibition (Suorsa et al., 2012; Huang et al., 2019a; Yamamoto and Shikanai, 2019; Tan et al., 2021). Therefore, we examined the performances of PSI and PSII under FL alternating between 59 and 1,809 μmol photons m^–2^ s^–1^ in young and mature leaves. The PSI parameters included Y(I) (the quantum yield of PSI photochemistry), Y(ND) (the oxidation ratio of P700), and Y(NA) {the extent of PSI over-reduction); and the PSII parameters included the quantum yield of PSII photochemistry (YII), non-photochemical quenching in PSII [Y(NPQ)], and quantum yield of non-regulatory energy dissipation in PSII [Y(NO)]}.

**FIGURE 2:**
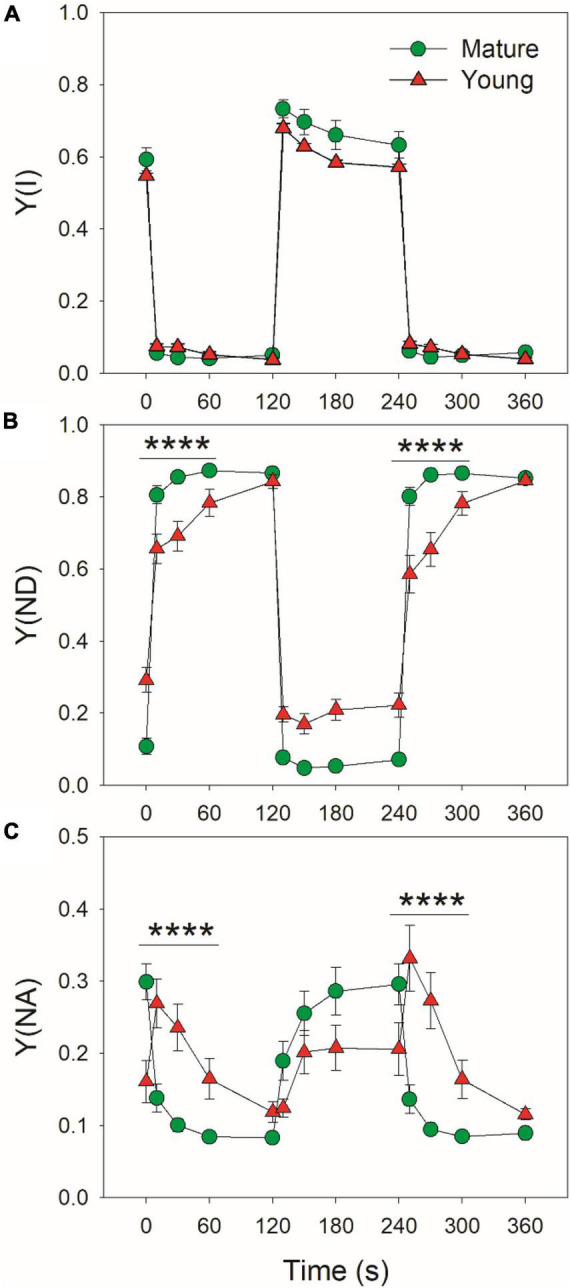
Changes in PSI parameters under fluctuating light alternating between 59 and 1809 μmol photons m^–2^ s^–1^ for mature and young leaves of Dendrobium officinale. **(A)** Y(I), the quantum yield of PSI photochemistry; **(B)** Y(ND), the oxidation ratio of P700; **(C)** Y(NA), the extent of PSI over-reduction. Data are means ± SE (*n* = 5). Asterisk indicates a significant difference between mature and young leaves.

At low light, mature leaves had similar Y(I) (Figure 2A), lower Y(ND) (Figure 2B), and higher Y(NA) (Figure 2C) when compared with young leaves. After transition to high light for 10 s, Y(ND) rapidly increased to high levels (>0.8) and Y(NA) rapidly decreased to low levels (<0.15) in mature leaves, indicating that PSI over-reduction was prevented in mature leaves when exposed to FL (Figures 2B,C). By comparison, Y(ND) increased more slowly in young leaves (Figure 2B). Concomitantly, Y(NA) abruptly increased to a peak in 10 s, followed by its gradual decrease, indicating the transient PSI over-reduction in young leaves under FL (Figure 2C). Therefore, the response of PSI redox state to FL largely differed between young and mature leaves.

**FIGURE 3:**
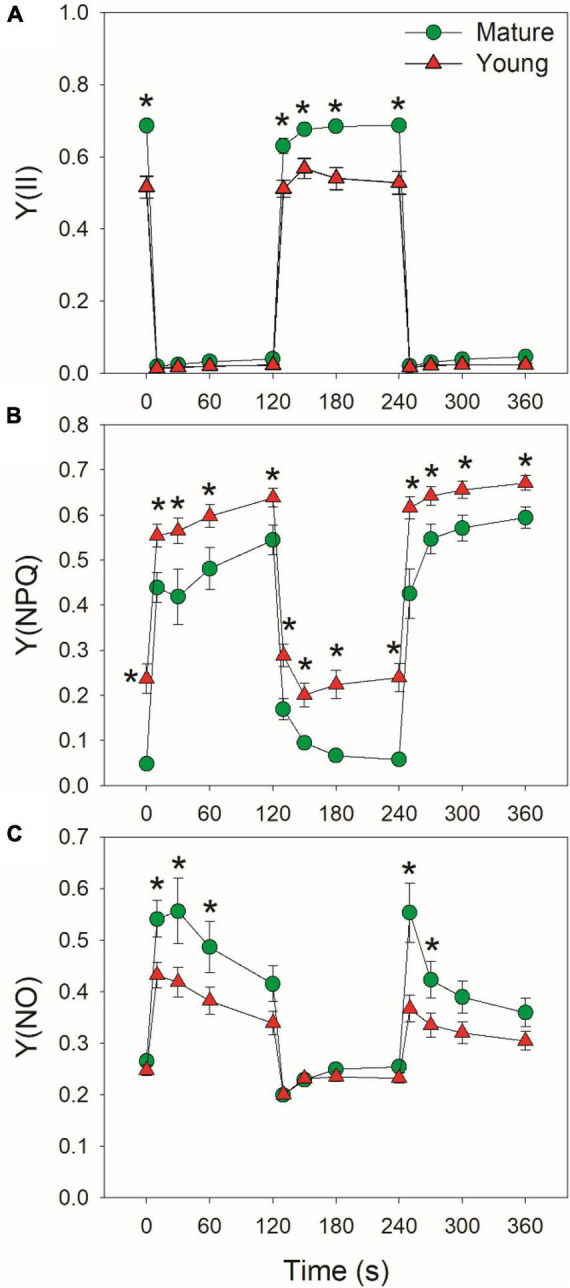
Changes in PSII parameters under fluctuating light alternating between 59 and 1809 μmol photons m^–2^ s^–1^ for mature and young leaves of Dendrobium officinale. **(A)** Y(II), the quantum yield of PSII photochemistry; **(B)** Y(NPQ), the quantum yield of non-photochemical quenching in PSII; **(C)** Y(NO), the quantum yield of non-regulatory energy dissipation in PSII. Data are means ± SE (*n* = 5). Asterisk indicates a significant difference between mature and young leaves.

At low light, mature leaves displayed higher Y(II), lower Y(NPQ), and similar Y(NO), when compared with young leaves (Figure 3), suggesting the lower light use efficiency in young leaves. After an abrupt increase in illumination, Y(II) largely decreased and Y(NPQ) gradually increased in mature and young leaves (Figures 3A,B). Concomitantly, Y(NO) first increased and then gradually decreased during the prolonged exposure to high light. The young leaves displayed higher Y(NPQ) capacity than mature leaves (Figure 3B), leading to lower Y(NO) under high light in young leaves (Figure 3C). The enhancement of Y(NPQ) in young leaves can dissipate the excess light energy harmlessly as heat and diminish the production of ROS. Therefore, young leaves upregulated NPQ to compensate the limitation of light use efficiency.

**FIGURE 4:**
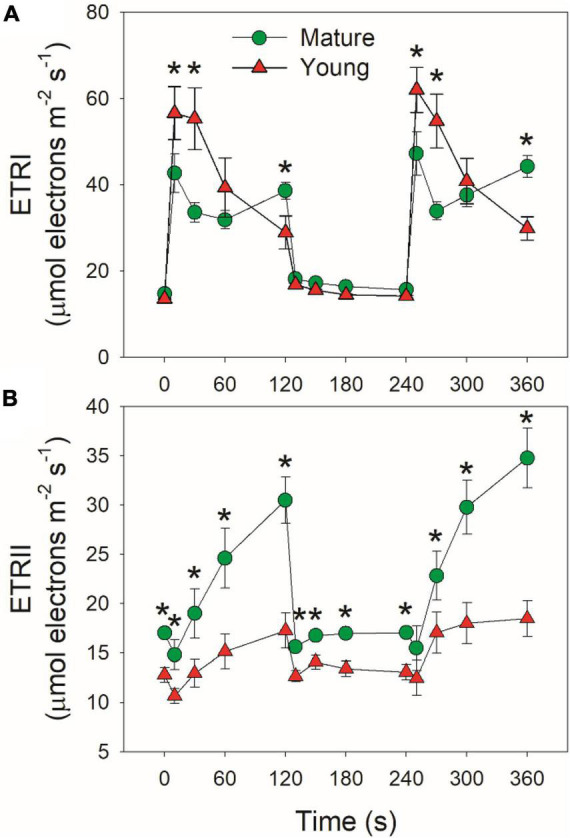
Changes in photosynthetic electron transport rates under fluctuating light alternating between 59 and 1809 μmol photons m^–2^ s^–1^ for mature and young leaves of Dendrobium officinale. **(A)** ETRI, electron transport rate through PSI; **(B)** ETRII, electron transport rate through PSII. Data are means ± SE (*n* = 5). Asterisk indicates a significant difference between mature and young leaves.

Mature and young leaves showed similar ETRI under low light (Figure 4A). Upon the transition to high light, ETRI rapidly increased within 10 s in mature leaves, followed by its decrease and re-increase (Figure 4A). By comparison, ETRI peaked in the first 10 s and then gradually decreased over time in young leaves. The performance of ETRII under FL was largely different from ETRI. By transitioning to high light, ETRII gradually increased in mature and young leaves (Figure 4B). After exposure to high light for 2 min, mature leaves displayed much higher ETRII than young leaves (Figure 4B). Since the operation of ETRII is largely determined by CO_2_ assimilation rate, this result indicates that under high light mature leaves have much higher CO_2_ assimilation rate than young leaves.

### Regulation of Cyclic Electron Flow Activation Under High Light

**FIGURE 5:**
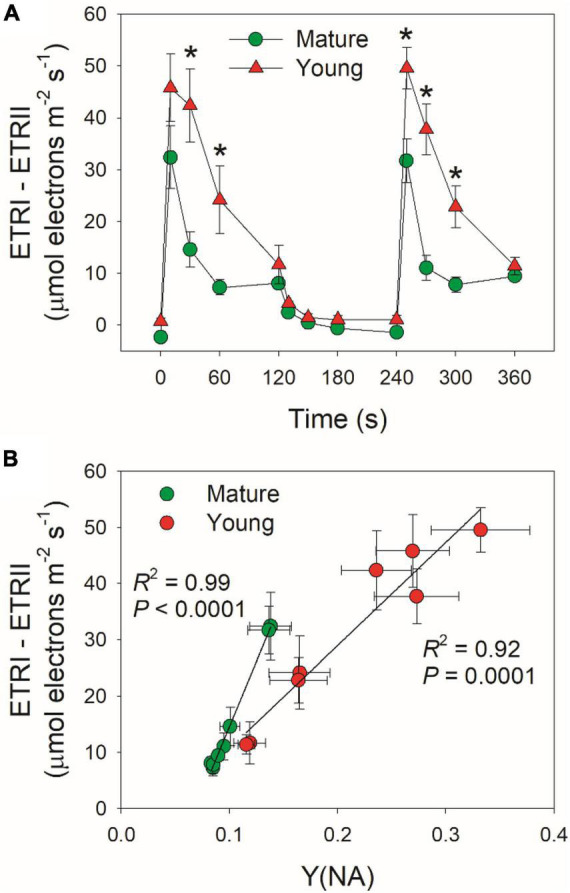
**(A)** Changes in ETRI–ETRII under FL alternating between 59 and 1,809 μmol photons m^–2^ s^–1^ for mature and young leaves of *D. officinale*. **(B)** The relationship between ETRI–ETRII and Y(NA) in high-light phases during FL. Data are means ± SE (*n* = 5). Asterisk indicates a significant difference between mature and young leaves.

Cyclic electron flow (CEF) contributes to the total photosynthetic electron transport and thus helps ΔpH formation (Wang et al., 2015; Shikanai and Yamamoto, 2017). Upon the transition to high light, ETRI–ETRII rapidly increased to the peaks in mature and young leaves within the first 10 s (Figure 5A). Subsequently, ETRI–ETRII gradually decreased in parallel. Because the difference between ETRI and ETRII is an indicator of CEF activation, these results indicated that CEF was highly activated within the first 10 s upon transition to high light. Furthermore, the CEF activation under FL was enhanced in young leaves than mature leaves. After this light transition for 2 min, ETRI–ETRII decreased to similar level in mature and young leaves. During this process, young leaves displayed much higher ETRI–ETRII values than mature leaves. Since an important role of CEF activation under FL is to alleviate PSI over-reduction, we examined the relationship between ETRI–ETRII and Y(NA), and found that the ETRI–ETRII value was strongly correlated to Y(NA) (Figure 5B). At the same ETRI–ETRII value, the Y(NA) was higher in young leaves than in mature leaves, indicating that young leaves enhanced CEF activity to protect PSI from the FL-induced over-reduction.

## Discussion

Generally, the induction speed of PSII electron flow is faster than that of CO_2_ assimilation in photosynthetic organisms, leading to the accumulation of excited states in PSI when light intensity abruptly changes from low to high (Gerotto et al., 2016; Yamori et al., 2016; Li et al., 2021). Meanwhile, photosynthetic angiosperms cannot generate a sufficient ΔpH (Huang et al., 2019a; Yang et al., 2021a), leading to a temporary uncontrolled electron flow from PSII to PSI through the Cyt b6f complex (Tikkanen and Aro, 2014; Armbruster et al., 2017). If the excess reducing power in PSI cannot be immediately consumed by downstream sinks of PSI, FL can induce a transient PSI over-reduction and thus causes PSI photoinhibition (Allahverdiyeva et al., 2013; Gerotto et al., 2016; Jokel et al., 2018; Yamamoto and Shikanai, 2019). To avoid FL-induced PSI photoinhibition, both flavodiiron proteins and CEF are employed by non-angiosperms to avoid PSI photoinhibition, in which flavodiiron proteins are the main players (Gerotto et al., 2016; Chaux et al., 2017; Shimakawa et al., 2017; Jokel et al., 2018). However, the genes of flavodiiron proteins are lacking in angiosperms (Ilík et al., 2017). Therefore, many angiosperms, such as *Arabidopsis*, rice, and tobacco display transient PSI over-reduction upon a sudden increase in irradiance (Yamamoto et al., 2016; Wada et al., 2018). Our results supported this notion by showing the transient increase in Y(NA) in young leaves after transition from low to high light (Figures 2A–C). To prevent an uncontrolled PSI over-reduction under high light, CEF around PSI is employed by angiosperms to help the rapid ΔpH formation (Suorsa et al., 2012; Kono et al., 2014; Tazoe et al., 2020). An increased ΔpH not only strengthens the downregulation of plastoquinone oxidation at the Cyt b6f but also enhances the electron sink downstream of PSI *via* providing additional ATP (Armbruster et al., 2017; Yamamoto and Shikanai, 2019). Consistently, we here observed the highly stimulation of CEF within the first 10 s after transition from low to high light in both young and mature leaves (Figure 5A). Additionally, an interesting phenomenon is that some angiosperms do not display PSI over-reduction under FL, which is caused by the operation of a pseudo-CEF pathway called WWC (Huang et al., 2019b; Sun et al., 2020; Yang et al., 2020). Therefore, angiosperms can use diverse strategies for protecting the PSI against FL-induced photoinhibition.

Both strategies are effective in protecting the PSI against photoinhibition under FL in angiosperms as demonstrated by their normal growth under natural FL conditions. However, CEF is a universal protective mechanism while the activity of WWC in angiosperms largely varies among angiosperms (Driever and Baker, 2011; Shirao et al., 2013; Huang et al., 2019b; Yang et al., 2020). The operation of WWC can consume excess light energy and favors the regulation of photosynthetic electron flow (Asada, 1999; Miyake and Yokota, 2000; Makino et al., 2002; Miyake, 2010; Alric and Johnson, 2017). The WWC activity in plants can be affected by environmental conditions, such as chilling temperature, drought stress, and high light (Zhou et al., 2004; Zivcak et al., 2013; Yi et al., 2014; Ferroni et al., 2021). It is unclear whether the activity of WWC is also affected by the ontogenetic stage of leaf in a given species. In the studied species *D. officinale*, WWC is documented to be operational in PSI photoprotection under FL in mature leaves. To test the effect of leaf ontogenetic stage on photosynthetic strategies coping with FL, the photosynthetic performance under FL was compared between mature and young leaves of *D. officinale*. We found that in mature leaves, WWC rapidly consumed excess reducing power in PSI and thus avoided the PSI over-reduction after any increase in illumination (Figure 1). In contrast, the WWC activity was negligible in young leaves as indicated by the clearly missing of rapid P700 re-oxidation upon transition from dark to actinic light. These results indicate that the establishment of WWC activity is largely dependent on the leaf ontogenetic stage. Furthermore, young leaves significantly displayed PSI over-reduction within the first 30 s after shifting from low to high light (Figure 2C), which was similar to the phenomenon observed in other angiosperms lacking WWC pathway (Yamamoto et al., 2016; Yamamoto and Shikanai, 2019). Therefore, the differential response of PSI to FL in mature and young leaves in *D. officinale* is largely caused by their difference in WWC activity.

It has been indicated that CEF and WWC have large functional overlap but can cooperate to protect PSI from photoinhibition under FL (Alboresi et al., 2019; Storti et al., 2019, 2020). In mature leaves of *D. officinale*, WWC was enhanced more strongly than CEF when exposed to FL at high temperature (Yang et al., 2021b). At low temperature, WWC activity was largely inhibited and CEF was highly activated to regulate the PSI redox state under FL (Huang et al., 2021). Upon the transition to high light at 25°C, WWC functioned to prevent the PSI over-reduction in the mature leaves. Meanwhile, CEF was stimulated moderately within the first 10 s. Therefore, WWC and CEF cooperate to fine-tune photosynthesis in mature leaves under FL at normal growth temperature (Sun et al., 2021). When light intensity abruptly shifted from low to high for 10 s, CEF was highly stimulated as indicated by the rapid increase of ETRI–ETRII value, and the CEF activation was stronger in young leaves than mature leaves (Figure 5A). Concomitantly, the PSI over-reduction was not completely avoided in young leaves. These results indicated that in young leaves, the lack of WWC activity was partially compensated by the enhancement of CEF. Therefore, mature and young leaves of *D. officinale* employed different strategies to adjust PSI redox state under FL. Furthermore, we observed positive relationship between CEF activation and PSI over-reduction (Figure 5B), suggesting that the CEF activation is affected by Y(NA). Compared with mature leaves, CEF was enhanced in young leaves to prevent the PSI over-reduction under FL. The PSI over-reduction indicates the insufficient ΔpH across the thylakoid membranes (Munekage et al., 2002, 2004; Yamamoto et al., 2016; Kanazawa et al., 2017; Takagi et al., 2017). Under such condition, the rapid stimulation of CEF helped ΔpH formation and thus prevented an uncontrolled PSI over-reduction in young leaves. By comparison, mature leaves mainly used WWC to prevent the PSI over-reduction and the major role of CEF was to balance ATP/NADPH production ratio *via* additional ATP synthesis. Therefore, the role of CEF in photosynthetic regulation under FL is flexible and can be affected by the operation of WWC.

In addition to the electron sink downstream, the redox state of PSI is affected by the PSII electron flow (Tikkanen et al., 2014; Suorsa et al., 2016; Terashima et al., 2021). At moderate PSII photoinhibition, the PSI over-reduction under high light is alleviated in *Arabidopsis pgr5* mutant (Tikkanen et al., 2014). Furthermore, the minimal activity of oxygen-evolving complex can rescue the lethal phenotype of *pgr5* when grown under FL (Suorsa et al., 2016). Therefore, when the capacity of CO_2_ assimilation rate is low, a low activity of oxygen-evolving complex is beneficial for protecting the PSI under FL. In young leaves of *D. officinale*, the maximum ETRII was much lower than mature leaves. Furthermore, the maximum value of Y(NA) under FL in young leaves was approximately 0.3, which was much lower than those in *Arabidopsis*, tobacco, and rice. These results indicated that the transient PSI over-reduction was slighter than the high-photosynthesis plants lacking WWC activity. Therefore, the relatively lower PSII activity in young leaves acts as a safety valve for alleviating the FL-induced PSI over-reduction.

## Conclusion

The response of PSI to FL varied among different plants and can be affected by environmental conditions. In this study, we examine the impacts of leaf ontogenetic stage on photosynthetic strategies used by *D. officinale* plants to cope with the FL. In mature leaves, WWC is mainly employed to avoid PSI over-reduction upon any increase in illumination. Concomitantly, CEF is stimulated to regulate the photosynthesis by adjusting the ATP/NADPH production ratio. In contrast, young leaves display PSI over-reduction under FL because WWC activity is absent. To compensate for the lacking of WWC activity, CEF is enhanced under FL to protect the PSI against photoinhibition. Therefore, the response of PSI to FL and the related photoprotective mechanisms are affected by leaf ontogenetic stage.

## Data Availability Statement

The original contributions presented in the study are included in the article/supplementary material, further inquiries can be directed to the corresponding authors.

## Author Contributions

WH and R-QM designed the study. Y-JY, QS, and HS performed the photosynthetic measurements. Y-JY, R-QM, and WH performed the data analysis. WH wrote first draft of the manuscript, which was extensively edited and approved the submitted version by all authors.

## Conflict of Interest

The authors declare that the research was conducted in the absence of any commercial or financial relationships that could be construed as a potential conflict of interest.

## Publisher’s Note

All claims expressed in this article are solely those of the authors and do not necessarily represent those of their affiliated organizations, or those of the publisher, the editors and the reviewers. Any product that may be evaluated in this article, or claim that may be made by its manufacturer, is not guaranteed or endorsed by the publisher.
